# Mechanism and Function of *Drosophila* capa GPCR: A Desiccation Stress-Responsive Receptor with Functional Homology to Human NeuromedinU Receptor

**DOI:** 10.1371/journal.pone.0029897

**Published:** 2012-01-11

**Authors:** Selim Terhzaz, Pablo Cabrero, Joris H. Robben, Jonathan C. Radford, Brian D. Hudson, Graeme Milligan, Julian A. T. Dow, Shireen-A. Davies

**Affiliations:** 1 Institute of Molecular, Cell and Systems Biology, College of Medical, Veterinary and Life Sciences, University of Glasgow, Glasgow, United Kingdom; 2 Department of Physiology, Nijmegen Centre for Molecular Life Sciences, Radboud University Nijmegen Medical Centre, Nijmegen, The Netherlands; University of Hong Kong, Hong Kong

## Abstract

The capa peptide receptor, capaR (*CG14575*), is a G-protein coupled receptor (GPCR) for the *D. melanogaster* capa neuropeptides, Drm-capa-1 and -2 (capa-1 and -2). To date, the capa peptide family constitutes the only known nitridergic peptides in insects, so the mechanisms and physiological function of ligand-receptor signalling of this peptide family are of interest. Capa peptide induces calcium signaling via capaR with EC_50_ values for capa-1 = 3.06 nM and capa-2 = 4.32 nM. capaR undergoes rapid desensitization, with internalization via a b-arrestin-2 mediated mechanism but is rapidly re-sensitized in the absence of capa-1. *Drosophila* capa peptides have a C-terminal -FPRXamide motif and insect-PRXamide peptides are evolutionarily related to vertebrate peptide neuromedinU (NMU). Potential agonist effects of human NMU-25 and the insect -PRLamides [*Drosophila* pyrokinins Drm-PK-1 (capa-3), Drm-PK-2 and hugin-gamma [hugg]] against capaR were investigated. NMU-25, but not hugg nor Drm-PK-2, increases intracellular calcium ([Ca^2+^]i) levels via capaR. *In vivo*, NMU-25 increases [Ca^2+^]i and fluid transport by the *Drosophila* Malpighian (renal) tubule. Ectopic expression of human NMU receptor 2 in tubules of transgenic flies results in increased [Ca^2+^]i and fluid transport. Finally, anti-porcine NMU-8 staining of larval CNS shows that the most highly immunoreactive cells are capa-producing neurons. These structural and functional data suggest that vertebrate NMU is a putative functional homolog of Drm-capa-1 and -2. capaR is almost exclusively expressed in tubule principal cells; cell-specific targeted capaR RNAi significantly reduces capa-1 stimulated [Ca^2+^]i and fluid transport. Adult capaR RNAi transgenic flies also display resistance to desiccation. Thus, capaR acts in the key fluid-transporting tissue to regulate responses to desiccation stress in the fly.

## Introduction

*Drosophila* is an excellent model for insect pest species especially the flies [1], [2]. As insects can withstand desiccation so well, in general, the detailed understanding of mechanisms of desiccation or water stress *in vivo* is a potential route for intervention. Insect neuropeptides, including diuretic peptides and their cognate receptors, are a key research area for potential novel routes for such control.

*D. melanogaster* capa- 1 and -2 (Drm-capa-1 and -2) neuropeptides act on the Malpighian tubules to increase fluid transport [3]. Tubules are transporting epithelia equivalent to vertebrate kidney and liver, and regulate water and ion homeostasis, and detoxification [4]. Capa-1 and capa-2 (and the related *Manduca sexta* CAP_2b_) are the only known nitridergic peptides in insects, acting via elevation of intracellular calcium, ([Ca^2+^]_i_) and activation of nitric oxide/cGMP signaling in tubule principal cells [3]. Capa peptides show a complex mode of action - in addition to stimulation of NO/cGMP signaling, capa-1 also modulates calcium signaling in the mitochondria [5], Golgi and peroxisomes [6]. There is also close conservation between capa peptide structure [7], [8] and capa-induced signaling cascades in tubules of the disease vectors *Anopheles*, *Aedes* and *Glossina* (tsetse fly) [9], [10]. Here, we demonstrate precise kinetics for capa-induced [Ca^2+^]_I_ signaling; and desensitization and internalization of capaR via b-arrestin.

The capa receptor (capaR) [11], [12] is a G-protein coupled receptor (GPCR) and member of the PRXamide peptide receptor family. The PRXamide C-terminal motif occurs in several invertebrate and vertebrate peptides [12]. There is significant interest in identification of vertebrate homologs of insect neuropeptides, as increasingly, key novel physiological functions *eg.*, in regulation of feeding behaviour, have been ascribed to insect neuropeptides [13]. Thus, potential homologous neuropeptide agonists of capaR are of significant interest. We identify human neuromedin U as a putative functional homolog of capa-1, via cell-based assays, *in vivo* assays using transgenic human NMU receptor 2 flies, and immunocytochemical studies in the larval nervous system.

The gene encoding capaR, *CG14575*, is highly expressed in *Drosophila* tubules; microarray analysis of *CG14575* gene expression in the tubule versus whole fly [14] demonstrates that *CG14575* is almost uniquely expressed in both of the adult and larval tubule. Given the importance of the tubule as a key tissue for homeostasis, it is possible that capaR has a significant role in organismal survival. A capaR promoter-specific GAL4 line allows expression mapping of endogenous capaR to tubule principal cells. Targeted expression of capaR RNAi in these cells decreases [Ca^2+^]_I_ under stimulation of capa-1 and abolishes capa-1 induced fluid transport. Finally, targeting of capaR RNAi to only tubule principal cells increases organismal survival to desiccation or water stress, demonstrating that capaR signaling in the tubule impacts on fluid homeostasis and on organismal survival.

## Materials and Methods

### *Drosophila* stocks and generation of transformants

All lines were maintained on a standard *Drosophila* diet at 22°C, 55% humidity on a 12∶12 h light∶dark photoperiod. Wild-type flies were obtained from a *Canton-S* (CS) stock. In order to drive cell- and tissue-specific gene expression of gene(s) of choice *in vivo*, the GAL4/UAS system was used, in which cell- or tissue-specific GAL4 ‘drivers’ enable binary expression of genes cloned downstream of the GAL4-binding Upstream Activating Sequence [15]. Thus, for intact tubules, principal cell-specific expression can be driven using either c42-GAL4 [16] or Urate-Oxidase-GAL4 [17] drivers in an otherwise normal fly. To assess *in vivo* calcium signals, doubly homozygous c42-GAL4>UAS-apoaequorin_cyto_ (c42aeq) flies were used, which specifically express the apoaequorin luminescent calcium reporter in the cytosol of the principal cells of the tubule main segment (upon which the diuretic neuropeptide capa-1 acts) [18]. The ubiquitous actin-GAL4 and the UAS-GFP lines were obtained from the Bloomington Stock Center (Bloomington, IN). To assess the impact of capaR and capaR RNAi on calcium signaling *in vivo*, lines were crossed to the doubly homozygous c42aeq flies. The ORF of the capaR (*CG14575*) was amplified from whole fly cDNA as template using the primers 5′-CGCGGCCGCATGAATTCATCGACCG-3′ and 5′-GCGGTACCTTAAATACAAGTCTC-3′ and cloned into the pUAST vector. To generate construct for heritable RNA interference (RNAi) of the capaR gene, an inverted repeat of a 615 base pair fragment was generated by PCR using the primers 5′-GCACTCTAGAACAAGGCAGTTTTGATAAC-3′ and 5′-GCACTCTAGAGTTCGAGATCGAATCTTGGC-3, and cloned as a tail-tail inverted repeat flanking the white intron into the P-element vector pWIZ [19]. Validation of the capaR RNAi line was confirmed by quantitative Q-RT-PCR using the primers 5′-GCTCTCCTTTGTGCGGGGGCACAT-3′ and 5′-GCACGTCAGAGCCAGCCAGCATCC-3′ (Fig. S1). To generate the capaR-GAL4 driver, the putative promotor sequence of the capaR gene was amplified by PCR using wild-type genomic DNA as a template with the primers 5′-CAGTCGACACCGGCAACCAC-3′and 5′-TTTAGCCCAGAGCTGAATGT-3′. The resulting amplicon (corresponding to bases −1 to −1501 from the transcriptional start site of the capaR coding region) was digested with KpnI and subcloned into pinGAL4 vector (gift of Dr. Jean-Christophe Billeter), which had previously digested with KpnI and CIP-treated. The ORF of the human neuromedin U receptor 2 (also referred to as FM4) was amplified from full-length cDNA clone (MHS1010-98075312, Thermo scientific) using specific primers: 5′-CACCATGTCAGGGATGGAAAAACTTC-3′ and 5′-TCAGGTTTTGTTAAAGTGGAAGCTTTG-3′. The ORF of the NMUR 2 gene was cloned into the pUAST vector using the Gateway system. The entry and destination vectors used were obtained from the *Drosophila* Gateway Vector collection (Invitrogen). All Transgenic lines were generated using standard methods for P-element-mediated germline transformation (BestGene Inc, USA).

### Plasmid construction for expression in *Drosophila* S2 cells

The ORF of capaR was amplified from whole fly cDNA as template using the primers 5′-GCGGTACCATGAATTCATCGACCG-3′ and 5′-GCGGTACCTTAAATACAAGTCTC-3′ and cloned into pMT/V5-His TOPO vector (Invitrogen). The eYFP ORF was fused in-frame at the C-terminus of the capaR using KpnI and ApaI sites and the tagged construct was cloned into the pMT/V5-His TOPO vector.

The construction of capaR-*Renilla* luciferase was realized by subcloning the full-length cDNA encoding *Renilla* luciferase (Rluc; 312 amino-acid) into a capaR-pMT/V5-His TOPO vector. The b-arrestin-2-eYFP pCDNA3 construct [20] was digested with KpnI and ApaI and subcloned into the pMT/V5-His TOPO vector. The ORF of the *CG8795* was amplified from fly cDNA using the primers 5′-ATGGCAGTCAAAATGCTGCCC-3′ and 5′-AAGGCGGCCCGCTCTTCA-3′ and was cloned into pMT/V5-His TOPO vector for expression in S2 cells. The ORF of the NMUR 2 gene was cloned into pMT/V5-His TOPO vector for expression in S2 cells.

### Peptide synthesis, peptide antibody production, immunofluoescence

The capa peptides Drm-capa-1 (GANMGLYAFPRVamide), Drm-capa-2 (ASGLVAFPRVamide), Drm-PK-1 (TGPSASSGLWFGPRLamide), Drm-PK-2 (SVPFKPRLamide) and hugin gamma (hugg, pQLQSNGEPAYRVRTPRL-amide) [3] were synthesised as C-terminally amidated peptides (Biomatik Corporation, Canada). Peptides were dissolved in distilled ACN/H_2_O to a concentration of 1 mM and then diluted to the required working concentration in Schneider's medium (Invitrogen Inc.). Human neuromedin U-25 was purchased from Sigma. Rabbit polyclonal antibody to porcine neuromedinU-8 was purchased from Progen biotechnik (Heidelberg, Germany). Rabbit capa precursor peptide used was described in [21] Rabbit anti-peptide antibody was raised against the capaR epitope (CQQGTNNRETRNSQM) by Genosphere Biotechnologies (Paris, France) and purified on a HiTrap NHS-activated HP column (Amersham Pharmacia Bio-tech; Buckinghamshire, UK) according to the manufacturer's instructions. Immunohistochemistry was carried out as described previously [21]. Mouse anti-GFP primary antibody (Zymed) and the antiserum to neuromedinU-8 were all diluted 1∶1000 or, in the case of the pre-immune serum, the affinity-purified capaR antibody and the antiserum to the capa precursor peptide, 1∶500. Incubations in the primary antibodies were performed overnight. A FITC-conjugated affinity-purified goat anti-mouse antibody (Jackson Immunologicals) was used in a dilution of 1∶1,000 for visualization of the mouse monoclonal anti-GFP. A Texas red-conjugated affinity-purified goat anti-rabbit antibody (Jackson Immunologicals) was used at a dilution of 1∶1,000 for visualization of the rabbit capaR antiserum.

For double labelling, larval brains were incubated with the antiserum to neuromedinU-8, which was visualised using a fluorescein-labeled F(ab) fragment of goat anti-rabbit IgG (Jackson Immunologicals), and subsequently with tetrarhodamine-labeled purified rabbit anti-capa precursor peptide serum [18]. For tubule immunohistochemistry, the nuclear stain DAPI (1 µg ml^−1^ for 1 min, Sigma) was used. The samples were cleared in a glycerol series (20%, 50%, and 80% glycerol/0.04 M Sodium Carbonate pH 9.4) and slides viewed using a Zeiss 510 META confocal microscope.

### Measurements of intracellular Ca^2+^ using aequorin

*Drosophila* S2 cells, cultured under standard conditions [21] were transiently transfected with the apoaequorin ORF [21] and a receptor ORF construct, and expression induced using CuSO4. Transfected S2 cells were harvested and incubated with 2.5 µM coelenterazine in the dark at RT for 1–2 h as previously described [21]. 25,000 cells were then placed in 135 µl Schneider's medium supplemented with 10% FCS in a well of a white polystyrene 96-well plate (Berthold Technologies). Bioluminescence recordings were carried out using a Mithras LB940 automated 96-well plate reader (Berthold Technologies) and MikroWin software. 15 µl of each of different peptides were applied to final concentrations as required. At the end of each recording samples were disrupted by the addition of 100 µl lysis solution, and the [Ca^2+^] concentrations calculated as previously described [18]. For assays in Ca^2+^-free medium, transfected cells were collected and pelleted after incubation with coelenterazine. Cell pellets were re-suspended in Ca^2+^-free Schneider's medium (Invitrogen Inc.) without FCS and collected by centrifugation. The washing and pelleting procedure was repeated once more. Experiments were then carried out on 25,000 cells per sample as above. To verify whether the observed calcium signal was linked to phospholipase C (PLC) activation, S2-capaR cells were preincubated with the PLC inhibitor U73122 at concentration of 10^−3^ M to 10^−8^ M for 10 min. Luminometry experiments were carried out on live, intact tubules expressing a targeted transgene for cytosolic-targeted apoaequorin in either principal or stellate cells as previously described [21], [22]. For *in vivo* tubule experiments, flies of the following genotypes were used: c42aeq, capaR-GAL4, UAS-capaR, UAS-capaR RNAi, UAS-NMUR 2 and the resulting progeny from GAL4>UAS crosses.

### Capa Receptor desensitization via b-arrestin

To assess the internalization and resensitization of capaR, *Drosophila* S2 cells were transiently transfected with the capaR-eYFP construct and either left untreated or treated with 10^−7^ M capa-1 for different times. Cells were subjected to immunocytochemistry with anti-GFP antibody, with assessment of fluorescence by confocal microscopy. For analysis of b-arrestin localization in response to capaR activation, S2 cells were transfected with b-arrestin-eYFP construct and real-time localization of b-arrestin-eYFP established by confocal microscopy in response to capa-1 stimulation.

### Cell Surface biotinylation and immunoblotting

For biotinylation experiments, S2 cells were left untreated or treated with 10^−7^ M capa-1 for 0, 5 10, 20 and 30 minutes to induce receptor internalization. An additional sample was incubated for 30 minutes with 10^−7^ M capa-1, washed three times with culture medium without capa-1 followed by 30 minutes incubation in culture medium to allow resensitization. Samples were rapidly cooled on ice followed by two washes with ice-cold PBS-CM. Cells were then subjected to cell surface biotinylation to label plasma membrane proteins [23] using EZ-Link Sulfo-NHS-SS-Biotin (Pierce). Total lysates and biotinylated samples (biotin-labelled protein was captured using streptavidin resin) were analyzed by SDS-PAGE (10% gel) followed by immunoblotting using affinity purified rabbit anti-capaR (1∶1000) as primary antibody according to standard techniques for S2 cells [24].

### Bioluminescence Resonance Energy Transfer (BRET) assay

S2 cells were co-transfected with capa receptor tagged with *Renilla* luciferase and b-arrestin-2 tagged with eYFP (ratio 1∶4), using calcium phosphate transfection method (Invitrogen). An additional transfection was performed with only the *Renilla* luciferase construct and empty expression vector pMT/V5-His TOPO vector. Cells were seeded at 200 000 cells per well into poly-D-lysine coated 96 well plates and coelentrazine-h (Promega, Southampton, UK) was added to a final concentration of 5 µM. Cells were incubated in darkness for 10 min at 37°C before addition of different concentrations of capa-1 peptide. Cells were incubated for a further 15 min at 37°C and subsequent BRET measurements were carried out using a PHERAstar FS reader (BMG-Labtech) that allows simultaneous reading of emission signals detected at 485 nm and 530 nm. The BRET ratio was then calculated as emission at 530 nm·emission^−1^ at 485 nm. Net BRET was defined as the 530 nm/485 nm ratio of cells co-expressing Rluc and eYFP minus the BRET ratio of cells expressing only the *Renilla* luciferase construct in the same experiment. This value was multiplied by 1000 to obtain mBRET units.

### Fluid transport assay by intact Malpighian tubules

Fluid transport assays were carried out as previously described [25] using live, intact tubules dissected from 7-day-old adults with the following genotypes: wild-type *Canton-S*, c42-GAL4>UAS-capaR RNAi, actin-GAL4>UAS-NMUR 2. Fluid droplets were collected every 10 min, and the volumes of fluid were calculated. Basal rates of fluid secretion were monitored for 30 min, whereupon peptide was added, and the secretion rate was then recorded for a further 30 min.

### Survival assays

5–7 day old flies of specified genotype were subjected to a starvation/desiccation stress in empty vials [26] in groups of approximately 30, with three biological replicates of each line. The tubule principal cell GAL4 driver (UO-GAL4) [16] was used to either over-express or knock-down capaR, with outcrossed (*Canton-S*) GAL4 and UAS lines. While the c42-GAL4 driver unequivocally drives expression in tubule principal cells in the adult and so is suitable for studies on acutely dissected adult tubules, it also directs expression in a few other tissues in the adult fly [27]. In order to assess the impact of tubule-targeted *capaR* transgenes on the survival of whole flies under starvation/desiccation stress, we used our Urate Oxidase-GAL4 driver (which directs expression only in the principal cells of both larval and adult tubule main segment), described in [17].

Before embarking on the desiccation survival assays, possible effects of the genetic background of these transgenic flies were avoided. Therefore, the UO-GAL4 and UAS-capaR/capaR RNAi lines used in this study were outcrossed for five generations in a White *Canton-S* background. In addition, the UAS-capaR/capaR RNAi and UO-GAL4 transgenic parental lines were crossed to White *Canton-S* (WhCS) and heterozygote progeny utilized to avoid the effect of 2 copies of the transgene.

Flies were counted until 100% mortality was reached and data expressed as % survival ± SEM (*N* = 3). Data were assessed for significance by the LogRank (Mantel-Cox) test using Graph Pad Prism 5.0 software. The number of flies used (N) was sufficiently high to allow for significant differences in survival; and the data were consistent between each of the 3 individual assays.

### Statistical analysis

Data are presented as mean ± S.E.M. Significance of differences was assessed with Student's *t*-test (two-tailed) for unpaired samples or one-way ANOVA, with significance taken as P<0.05, marked graphically with an asterisk.

## Results and Discussion

### Capa receptor-associated calcium signatures

**Figure 1:**
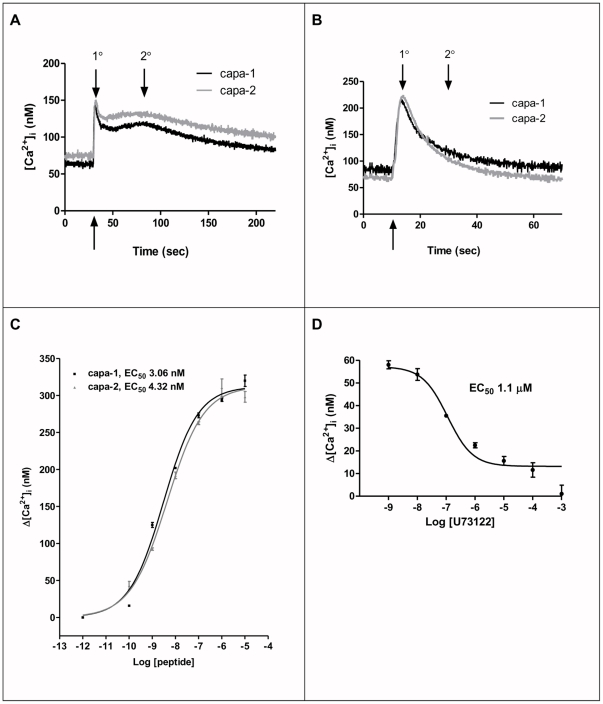
[Ca^2+^]_i_ signatures in response to capa-1 and capa-2 in capaR- and apoaequorin-transfected S2 cells. (**A**) [Ca^2+^]_i_ increases stimulated by 10^−7^ M capa-1 (black) and capa-2 (grey). The traces shown are typical data from single experiments. Data are expressed as [Ca^2+^]_i_ (nM) against time (s); each data point corresponds to 0.1 s; agonist injection indicated by an arrow. (**B**) Real-time measurement of the [Ca^2+^]_i_ response to the capa peptides under Ca^2+^-free conditions. Data are expressed as [Ca^2+^]_i_ (nM) against time (s); each data point corresponds to 0.1 s. The graphs display [Ca^2+^]_i_ increases in capaR and apoaequorin expressing S2 cells. Traces shown are typical data from single experiments in response to 10^−7^ M capa-1 (black) or capa-2 (grey) in the absence of Ca^2+^. In (**A**) and (**B**) the upward arrow indicate the time of peptide agonist injection while the downward arrows indicate the primary and secondary capa-induced calcium responses. (**C**) Dose-response curves for capa-1 and capa-2. Action of the capa peptides (capa-1 (black) or capa-2 (grey)) on S2 cells expressing capaR and apoaequorin. Values were expressed as maximal (nM) - background (nM) (mean ± S.E.M., *N* = 6). Where error bars are not visible they are too small to reproduce. (**D**) Concentration-response for the action of the PLC inhibitor U73122. Cells were challenged with increasing concentrations of U73122, and calcium mobilization was measured. Values were expressed as maximal (nM) - background (nM) (mean ± S.E.M., *N* = 3).

In *Drosophila* S2 cells assays, *CG14575* encodes a functional receptor for both the capa-1 and capa-2 peptides [11], [12] (Fig. 1A). Stimulation of capaR with both capa-1 and capa-2 results in a biphasic rise in [Ca^2+^]_i_, comprising a rapid primary peak followed by a slower secondary peak. The secondary [Ca^2+^]_i_ response is abolished when external Ca^2+^ is removed (Fig. 1B).

*CG14575* responds to both capa-1 and capa-2 in a dose-dependent manner (Fig. 1C). EC_50_ values for stimulated [Ca^2+^]_i_ responses for capa-1 and capa-2 in the nM range (3.06 nM, 4.32 nM respectively). *CG14575*-encoded receptor also responds to the lepidopteran peptide CAP_2b_ [28], a member of the capa family (data not shown). Previous work on *CG14575* - transfected CHO cells [11] and *Xenopus* oocytes [12] show values in the 10^−7^ M range. Here we show Capa-induced [Ca^2+^]_i_ increases are similar in size and dynamics to the response seen in principal cells of intact Malpighian tubules [3], (Table S1), with EC_50_ values at nM concentration.

In intact tubules, removal of external calcium, or pharmacological/genetic intervention of plasma membrane channels including L-type, CNG and TRP/TRPL channels significantly reduce or even abolish CAP_2b_/capa-stimulated [Ca^2+^]_i_ responses [29]. The primary [Ca^2+^]_i_ response is due to release from internal Ca^2+^ stores (Fig. 1B) via the Phospholipase C (PLC)/Ins 1,4,5 trisphosphate (InsP_3_) pathway [30] (Fig. 1D), where the capa-1-induced [Ca^2+^]_i_ response is inhibited by the widely-used PLC inhibitor (U73122) in a dose-dependent manner.

Overall, these S2 cell data are consistent with the role of capa-sensitive intracellular calcium channels and of the role of influx of extracellular calcium via plasma membrane calcium channels in the tubule.

### Desensitization and internalization of the capaR

**Figure 2:**
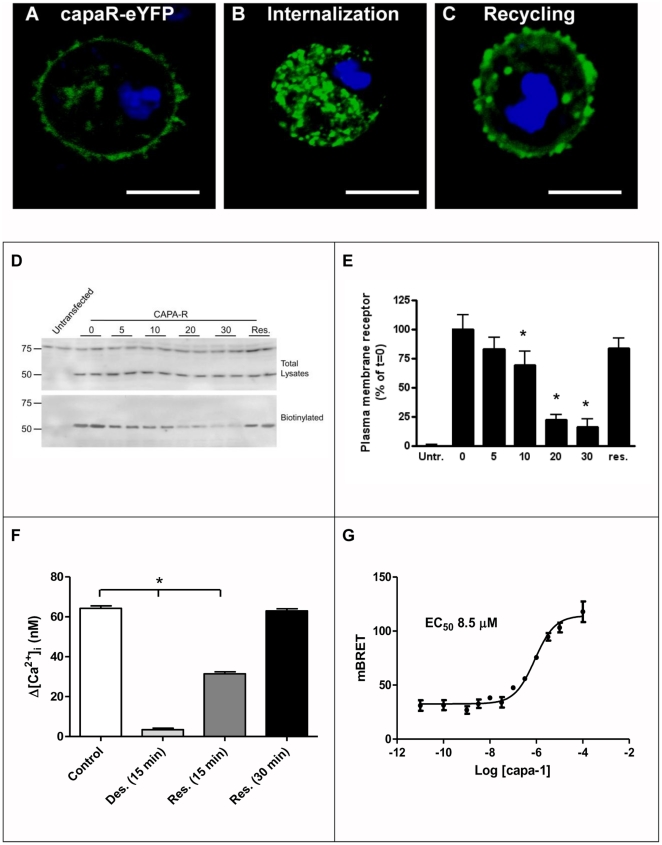
Desensitization and internalisation of capa-1-stimulated capaR. S2 cells were transfected with eYFP-tagged capaR, left un-treated or treated with capa-1, and viewed by confocal microscopy after immunocytochemistry with anti-GFP antibody. (**A**) Control. (**B**) capa-1 stimulated, 15 min. (**C**) sample was incubated for 15 minutes with capa-1, washed three times with culture medium followed by 30 minutes incubation in culture medium to allow resensitization. Nuclei are labelled blue with DAPI, scale bar represents 10 µM. (**D**) S2 cells expressing capaR were left untreated (0), or treated for 5, 10, 20 or 30 minutes (indicated) with 10^−7^ M capa-1 to induce receptor internalization. An additional sample was incubated for 30 minutes with capa-1, washed three times with culture medium without capa-1 followed by 30 minutes incubation in culture medium to allow resensitization (Res.). A sample of untransfected cells serves as a negative control. Samples were subjected to cell surface biotinylation to label plasma membrane proteins. We found that the protein concentration of biotinylated samples are generally lower than that of the total lysates; therefore, the equivalent of 5000 cells were loaded for the total lysate, and an equivalent of 15,000 cells were loaded for the biotinylated samples. Total lysates and biotinylated samples were subjected to western blot analysis. Immunoblot using anti-capaR antibody identified a band of the predicted size of 52 kDa which confirms the specificity of the antibody and an additional non specific 75 kDa protein absent in the cell-surface (biotinylated) fraction. (**E**) Samples from the cell surface biotinylation experiment were semi-quantified and corrected for total receptor expression. Relative cell surface expression is shown as a percentage of the non-treated S2 cells expressing capaR (t = 0). Bars indicated with an asterisk were significantly (P<0.05 as determined by one-way ANOVA) reduced compared to t = 0. (**F**) Calcium measurements in S2 cells transfected with expression constructs for aequorin and the capa receptor. S2 cells were challenged with 10^−7^ M capa-1, pre-treated with capa-1 for 15 min (Desensitization (Des.)), followed by ligand removal after which S2 cells were challenged at 15 min or 30 min (Resensitization (Res.)) with 10^−7^ M capa-1 and cytosolic [Ca^2+^]_i_ levels measured. Bars indicated with an asterisk were significantly (P<0.05 as determined by Student's *t*-test) reduced compared to control. (**G**) Analysis of capaR-β-arrestin-2 interactions. S2 cells were co-transfected with capa receptor tagged with *Renilla* luciferase and β-arrestin-2 tagged with eYFP. Bioluminescence Resonance Energy Transfer (BRET) signals were monitored after treatment of the cells for 15 min with varying concentrations of capa-1. Data are expressed as mBRET units ± SEM, *N* = 3.

GPCRs undergo desensitization and/or internalization shortly after agonist stimulation. eYFP-tagged capaR-transfected S2 cells were used to address the internalization/recycling process for capaR (Fig. 2A–C). Capa-1 treatment results in internalization (intracellular vesicles) (Fig. 2B) after which endocytic recycling to the plama membrane occurs during resensitization in the absence of capa-1 (Fig. 2C).

Cell-surface biotinylation experiments can reliably indicate the status of receptors and membrane-associated proteins as this technique permits the labeling and purification of plasma membrane proteins [31]. In resting capaR transfected S2 cells, the capaR signal is very strong in the biotinylated fraction indicating high level of capaR at the plasma membrane, but upon stimulation with 10^−7^ M capa-1, the cell-surface (biotinylated) fraction was reduced and quickly restored upon removal of the agonist (Fig. 2D). Quantification of plasma membrane-localized capaR (Fig. 2E) showed that the capaR signal is significantly reduced during desensitization, due to internalization, and then recycled to the plama membrane during resensitization.

To what extent is capaR signalling affected during internalization/recycling? [Ca^2+^]_i_ measurements showed that pre-treatment of S2-capaR cells with 10^−7^ M capa-1 for 15 min completely abolished the capa-1 induced calcium response, indicating desensitization of the capaR (Fig. 2F). After ligand removal, S2 cells were challenged at 15 min or 30 min with 10^−7^ M capa-1 and cytoslic [Ca^2+^]_i_ levels measured. A 15-minute resensitization period sees a modest capa-1 [Ca^2+^]_i_ response and a response similar to control at 30 min (Fig. 2F), correlating with the % of plasma membrane-localized capaR (Fig. 2E).

GPCRs are desensitized by a GPCR Regulating Kinase (GRK)/arrestin-mediated mechanism. In *Drosophila*, two GRKs [32] and a non-visual arrestin [33] have been identified. Desensitization of capaR involves a GRK-associated mechanism, as capa-1 induces increased *GRK-2* gene expression in tubules (Fig. S2). Desensitization of insect [34] and mammalian GPCRs can also be assessed by translocation of b-arrestin in transfected mammalian cells [35]. Video S1 demonstrates the translocation of cytosolic– localized mammalian b-arrestin2-eYFP to the membrane in response to capa-1 in capaR-transfected S2 cells. To further demonstrate the direct capaR-b-arrestin-2 interaction, Bioluminescence Resonance Energy Transfer (BRET)-based b-arrestin-2 interaction assays [36] were performed by co-transfecting S2 cells with capaR-C-terminally tagged with *Renilla* luciferase and b-arrestin-2-eYFP. Capa-1 produced a clear concentration-dependent increase in BRET, reflecting capaR-b-arrestin-2 interactions, with EC_50_ = 8.5 µM (Fig. 2G).

Agonist-induced activation of GPCRs plays a major role in the regulation of signal transduction pathways, either by propagating or terminating signals. Our data support the hypothesis that capa-1 release regulates capaR activation and subsequent internalization, thereby also contributing to signal termination (unless the internalized receptor continues signalling). Can the physiological relevance of capaR activation and desensitization be explained by the function of the peptides? The *Drosophila* capa peptides have been shown to increase fluid secretion by the Malpighian tubules. However, insects generally need to retain water as much as possible; this could be achieved by releasing the capa peptides at the right physiological moment (e.g., during feeding) and once feeding is over, the diuretic action of the capa peptides needs to be terminated for the insect to conserve water. The rapid desensitization of the capaR may limit the responsiveness of the receptor to repeat agonist challenge and the physiological consequence would be to limit water loss.

### Is Neuromedin U a putative functional homolog of capa peptide *in vivo*?

**Figure 3:**
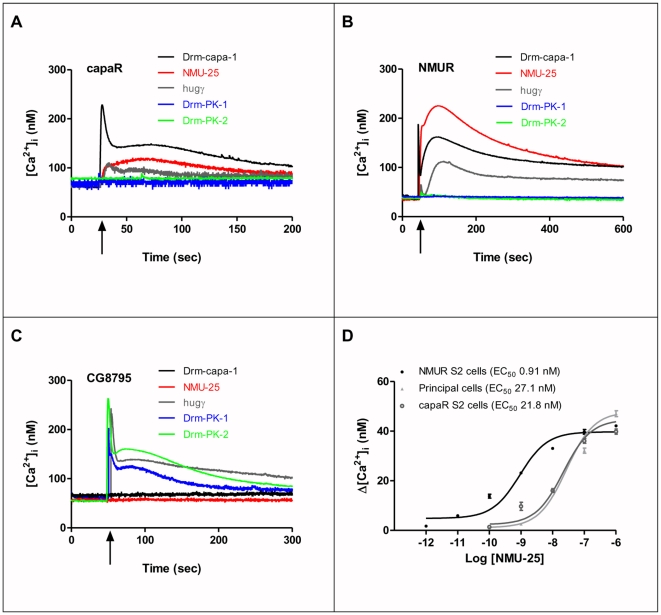
Capa, Neuromedin and Hugin receptor *(CG8795)*-associated calcium signatures. (**A**) Typical cytoplasmic Ca^2+^ response in S2 cells expressing capaR and apoaequorin challenged with Drm-capa-1, Drm-PK-1, hugg, Drm-PK-2 and NMU-25 at a concentration of 10^−7^ M. (**B**) Typical cytoplasmic Ca^2+^ response in S2 cells expressing NMUR 2 and apoaequorin challenged with Drm-capa-1, Drm-PK-1, hugg, Drm-PK-2 and NMU-25 at a concentration of 10^−7^ M. (**C**) Typical cytoplasmic Ca^2+^ response in S2 cells expressing *CG8795*and apoaequorin challenged with Drm-capa-1, Drm-PK-1, hugg, Drm-PK-2 and NMU-25 at a concentration of 10^−7^ M. (**D**) Human NMU-25 dose-response curve in S2 cells and intact tubule. NMU-25 peptide stimulation of NMUR 2- or capaR- and apoaequorin-co-transfected S2 cells; and of tubule principal cells expressing apoaequorin transgene. Cells or tubules were challenged with increasing concentrations of agonist, and [Ca^2+^]_i_ was measured. Values were expressed as maximal (nM) - background (nM) (mean ± S.E.M., *N* = 3).

The amino acid sequence of human neuromedinU-25 (NMU-25) shows evident homology to capa peptides [12] (Table 1). We thus investigated the possibility that mammalian NMU can act as an agonist for capaR. To validate functional human NMU receptor 2 (NMUR 2) in S2 cells, [Ca^2+^]_i_ was measured in human NMU-25 stimulated NMUR 2- and apoaequorin-co-transfected S2 cells (Fig. 3A). NMU-25 has a small but significant effect on [Ca^2+^]_I_ via capaR (Fig. 3A). We then tested the action of capa-1 at the NMU receptor 2 and show that capa-1 mobilizes [Ca^2+^]_i_ via NMUR 2 (Fig. 3B). Based on structural and functional similarities, *Drosophila hugin* has been proposed as an homolog of mammalian NMU [37]. *Hugin* encodes two peptides: hugin gamma (hugg) and Drm-PK-2, whose C-terminal motifs are related to the insect pyrokinins. Our data show that hugg increases [Ca^2+^]_i_ via NMUR 2 but to a reduced level compared to capa-1. Interestingly, Drm-PK-1 (capa-3) and -2 do not have effects on either capaR (Fig. 3A) or NMUR 2 (Fig. 3B). However, as Drm-PK-1 and -2 share the -PRLamide signature with hugg (Table 1), we tested their action on *CG8795*. Stimulation of *CG8795*- and apoaequorin-co-transfected S2 cells with Drm-PK-1, - 2, and hugg peptides (Fig. 3C), stimulates increased [Ca^2+^]_i_ levels. However, neither capa-1 nor NMU-25 acts via *CG8795*.

**Table 1:** 

Peptide	Sequence
Human NMU-25	FRVDEEFQSPFASQSRGYFLFRPRN-amide
Porcine NMU-8	YFLFRPRN-amide
Drm-capa-1	GANMGLYAFPRV-amide
Drm-capa-2	ASGLVAFPRV-amide
Aplysia SCPB	MNYLAFPRM-amide
Drm-PK-1 (capa-3)	TGPSASSGLWFGPRL-amide
Drm-PK-2	SVPFKPRL-amide
hugg	pQLQSNGEPAYRVRTPRL-amide

The absence of NMU agonism to *CG8795* receptor has been demonstrated [38] and while the homology of NMU to pyrokinins and their respective receptors are higher compared to the capa family of peptides [7], [11], [35], Drm-PK-2 does not activate the NMUR 2, in contrast to capa-1. Moreover, Fujii et *al.*, have found that *Aplysia* small cardioactive peptide B (SCPB), which shares the consensus motif, L*XX*PR*X*-amide, with neuromedin U, shows a significant agonistic activity to NMR 1 expressed in CHO cells [39]. This indicates that structurally related capa-like peptides, unlike pyrokinins, are able to activate vertebrate NMURs and therefore represent functional NMU homologous. We next investigated a potential role for NMU by challenging, with different concentrations of NMU-25, capaR transfected S2 cells or acutely dissected intact Malpighian tubule. Calcium measurements using NMUR 2-transfected S2 cells expressing the apoaequorin transgene (Fig. 3D) showed a robust dose-response curve with an EC_50_ = 0.91 nM, which is within the nM range obtained with NMUR 2 in HEK293 cells [40]. Furthermore, transgenic tubules in which targeted apoaequorin is expressed in tubule principal cells showed a dose-dependent response to NMU-25 with an EC_50_ = 21.8 and 27.1 nM respectively (Fig. 3D) demonstrating that NMU does indeed act on tubules, via endogenous capaR (Fig. 3A).

**Figure 4:**
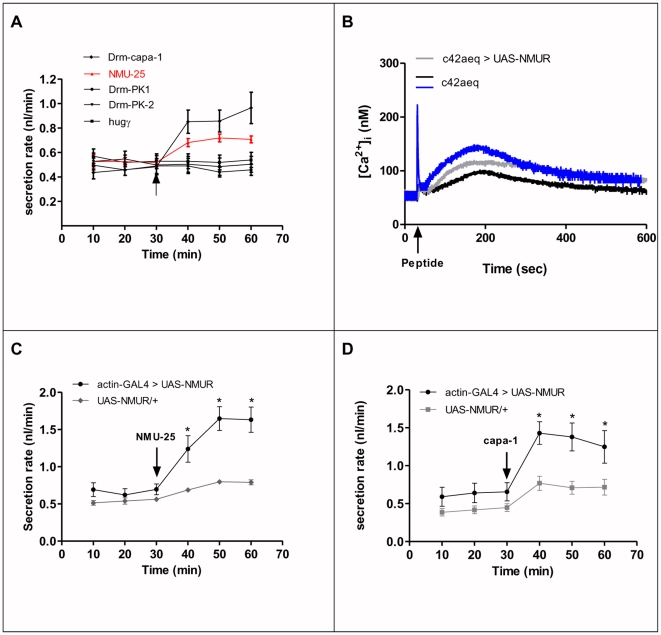
Functional role of NMU *in vivo*. (**A**) Fluid transport by *Drosophila* wild-type tubule is significantly increased after application of the peptides Drm-capa-1 and NMU-25 at 10^−7^ M but not with Drm-PK-1, hugg, Drm-PK-2. (**B**) [Ca^2+^]_i_ levels in NMU-25-stimulated (10^−7^ M) transgenic tubules expressing UAS-NMUR 2 and apoaequorin transgenes driven by c42-GAL4 (grey) compared with a typical control response (black, c42aeq). The calcium trace in blue represent a typical biphasic capa-1 (10^−7^ M) response in tubule principal cells. Pooled cytosolic [Ca^2+^] data from separate experiments are shown where data are nM [Ca^2+^] ± SEM, *N* = 6, where *P*<0.05. (**C**) NMU-25 (10^−7^ M) stimulates increased fluid transport in transgenic tubules, in which UAS-NMUR 2 was driven by actin-GAL4. (**D**) capa-1 (10^−7^ M) stimulates increased fluid transport in transgenic tubules, in which UAS-NMUR 2 was driven by actin-GAL4. Data are expressed as mean fluid transport rate (nl/min) ± SEM, N = 6–10. The level of significance in A, C and D was determined using a Student's *t*-test (* P<0.05) and in C and D, statistical analysis was confined to the comparison between the parental and progeny response.

Given that NMU directly activates capaR, we tested the possibility that NMU could have functional roles *in vivo*. Fluid transport in wild-type tubules is significantly increased by human NMU-25 but not by Drm-PK-1 and -2, or hugg (Fig. 4A). Using transgenic lines for human NMUR 2, in which ectopic expression in tubule was achieved under control of the principal cell-specific c42-GAL4 driver, we show that NMU increases [Ca^2+^]_i_ *in vivo* (Fig. 4B). Ectopic expression of human NMUR 2 in tubules using the ubiquitous actin-GAL4 driver also results in significantly elevated fluid transport upon NUM-25 stimulation (Fig. 4C); presumably due to increased [Ca^2+^]_i_ in principal cells (Fig. 4B). Capa-1 stimulation of NMUR 2 tubules also increases fluid transport rates (Fig. 4D), suggesting that the action of capa-1 at the NMUR 2 receptor (Fig. 3B) can have a physiological role *in vivo*.

**Figure 5:**
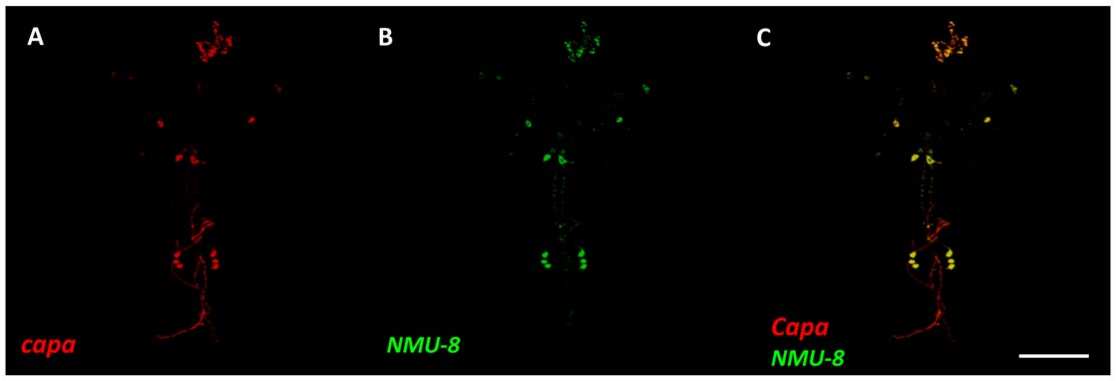
Immunocytochemical localization of the capa-expressing neurons with a vertebrate NMU antibody. Larval central nervous system was doubly labeled using an antibody against capa precursor (**A**, red), an antibody against porcine neuromedinU-8 (**B**, green). The immunoreactive cells bodies and neurohemal organs, the retrocerebral complex for the 2 cell bodies in the subesophageal neuromere, and the abdominal median transverse nerves for the 3 pairs of abdominal neuroendocrine cells all co-localize (**C**, yellow, merge). Scale bar 100 µm.

Based on the sequence similarities between capa, hugin and NMU peptides, we performed immunocytochemistry using anti-neuromedinU-8 antibody on larval brain. The most strongly immunoreactive cells in the nervous system recognized by the neuromedinU-8 antibody consist of three pairs of ventral neuroendocrine cells in the abdominal neuromeres and a single pair of very large neuroendocrine cells in the subesophagial ganglion (Fig. 5). These immunoreactive cells are identical to the capa neuroendocrine cells [3] demonstrated using anti-capa precursor peptide antibody. It is worth mentioning that anti-NMU-8 also labels hugin neurons (small group of neurons in the SOG) but in a lower extent. Taken together, these data suggest that NMU could be regarded as a functional vertebrate homolog of capa-1. In vertebrates, NMU action modulates several physiological processes feeding and the stress response, as NMU interacts anatomically and functionally with the CRH system which is secreted in response to stressors [41]. There is some evidence that NMU can affect ion transport in the gut [41], but this is the first evidence that NMU can modulate fluid transport by a renal system.

### Physiological role of capaR *in vivo*

**Figure 6:**
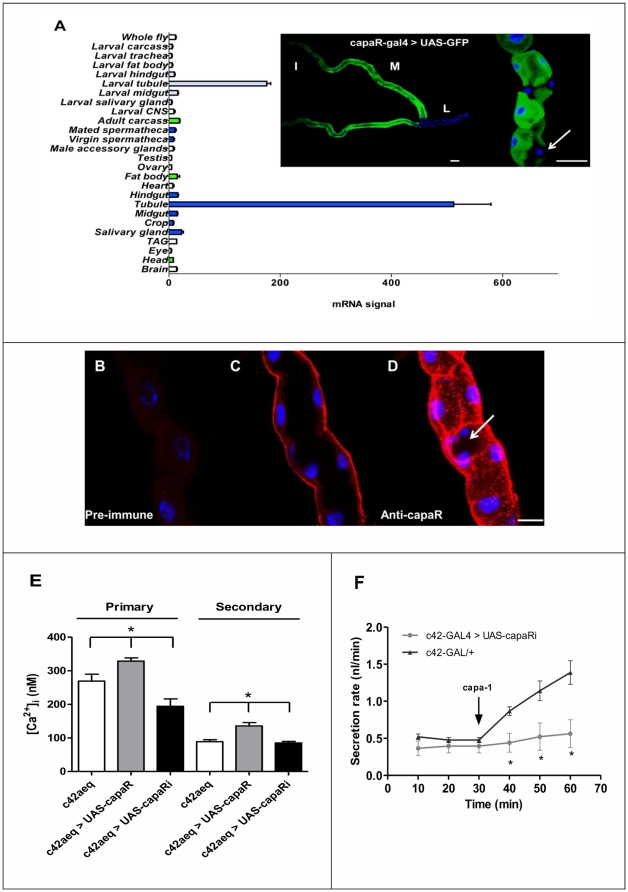
capaR is tubule-specific and localized to principal cells; manipulation of capaR expression levels modulates [Ca^2+^]_i_ and fluid transport rates. (**A**) Mean mRNA expression data ± SEM were collated from Affymetrix tissue-specific array datasets described in flyatlas.org [14] for adult and larval tissues as indicated. Blue shading (dark-adult; light-larvae) indicates epithelial tissues; whereas green shading (dark-adult; light-larvae) indicates fat body or tissues containing fat body *eg.*, adult head and carcass. ‘mRNA signal’ indicates how abundant *capaR* mRNA is; and for each tissue, *capaR* mRNA was detectably expressed in 4 out of 4 arrays (flyatlas.org). In order to assess the expression pattern of capaR *in vivo*, the capaR promoter-driven GAL4 line, capaR-GAL4, was generated and crossed with UAS-GFP, and fluorescence examined by GFP histochemistry in tissues from progeny of the cross (top left panel). For orientation, tubule regions are indicated by M (main segment); I (initial segment); L (lower tubule). Expression of capaR-driven GFP occurs in the principal cells in the tubule main segment, exclusion of a stellate cell (arrowed, top right panel). (**B–D**) *Drosophila* capa receptor is expressed in principal cells of the Malpighian tubule. (**B**) Tubules were processed with pre-immune serum and only low-level non-specific staining of intracellular vesicles was observed, confirming the specificity of the antibody. (**C**) Immunocytochemistry using anti-capaR rabbit polyclonal antibody and anti-Rabbit IgG-Texas Red conjugate reveal basolateral membrane localization of capaR in tubule principal cells. (**D**) Merge of z-stacks from (**B**) picture reveals exclusion of a stellate cell (arrowed). In panels A, B–D, nuclei are labelled blue with DAPI, scale bar represents 30 µM. (**E**) Manipulation of capaR affects cytosolic [Ca^2+^]_i_ levels in intact tubules. Tubules were dissected from c42>UAS-apoaequorin flies (c42aeq), c42aeq>UAS-capaR RNAi flies and c42aeq>UAS-capaR. Resting cytoslic [Ca^2+^]_i_ levels were measured, after which tubules were stimulated with 10^−7^ M capa-1 to obtain stimulated cytosolic [Ca^2+^]_i_ readings. Primary and secondary pooled data for cytosolic [Ca^2+^]_i_ levels are shown as nM [Ca^2+^]_i_ ± SEM, *N* = 6, where * P<0.05, Student's *t*-test. (**F**) Fluid transport by *Drosophila* c42-GAL4>capaR RNAi renal tubules is significantly decreased (as determined using a Student's *t*-test (*P<0.05)) compared to the parental GAL4 line when the tubule is stimulated by application of capa-1 (10^−7^ M). Secretion rates are expressed as nl/min ± SEM (*N* = 6).

The capaR gene is expressed exclusively in Malpighian tubules in both larvae and adults (Fig. 6A), where capaR expression is up-regulated 42- (adult) and 14.4- fold (larvae) in tubules compared to the whole fly. The putative control region of the capaR gene drives expression of GAL4 in the capaR-GAL4 line: progeny from a cross of capaR-GAL4 and a UAS-GFP line show that fluorescence was specifically detected in the Malpighian tubules, either at the third instar larval or adult stages (Fig. 6B), a result consistent with the microarray data for capaR expression (Fig. 6A). Furthermore, tubule-specific capaR is expressed at the basolateral membrane of the principal and not stellate cells (Fig. 6B–D). Also, typical capa-1-induced Ca^2+^ responses occur in principal cells of capaR-GAL4>UAS-apoaequorin_cyto_ transgenic tubules but Ca^2+^ responses do not occur in response to stellate-cell specific drosokinin [21] (data not shown). Taken together, these data are consistent with the action of capa peptides on only principal cells of the tubule [3] and also confirm the exquisite principal-cell specificity of the capaR GAL4 driver. Manipulation of capaR expression levels in only the tubule principal cells results in direct modulation of capa-induced calcium and fluid-transport rates (Fig. 6E, 6F). Targeted over-expression of capaR to only tubule principal cells results in increased [Ca^2+^]_i_; whilst a cell-specific knockdown of capaR RNAi (Fig. S1) results in significantly decreased capa-1-stimulated [Ca^2+^]_i_, and fluid transport. Thus, modulation of expression of this single GPCR *in vivo* has physiological consequences for epithelial function.

**Figure 7:**
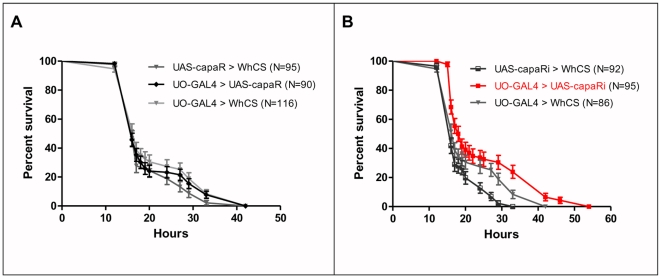
Knock-down of capaR expression in principal cells enhances organismal survival to desiccation stress. (**A**) Increased capaR levels in principal cells (UO-GAL4>UAS-capaR, black line) do not alter survival of desiccated flies. (**B**) Reduced capaR levels in principal cells (UO-GAL4>UAS-capaRi, red line) alter survival of desiccated flies. Desiccation resistance was significantly higher after knockdown of capa receptor in principal cells compared to controls (P<0.001 against both controls; Log rank test, Mantel-Cox).

As capaR impacts so critically on fluid transport, we investigated the role of the capaR in starvation/desiccation stress. The tubule principal cell GAL4 driver (UO-GAL4) [17] was used to either over-express or knock-down capaR, with outcrossed (*Canton-S*) GAL4 and UAS lines. Perhaps unsurprisingly, over-expression of capaR (Fig. 7A) or of NMUR 2 (data not shown) did not affect survival under these stress conditions. The effect of over-expressing capaR in tubule principal cells does not impact critically on calcium signalling and fluid secretion, so may not impact on the physiological response to desiccation stress. Furthermore, we have found that lower levels of capa peptides are released during desiccation (data not shown) so increasing the levels of capaR will not necessarily make any difference to the desiccation stress response.

By contrast, flies with reduced capaR levels exhibited significantly extended survival compared to controls under starvation/desiccation stress (Fig. 7B) (P<0.001 against both controls; Logrank test, Mantel-Cox), presumably due to reduced fluid loss by the tubule, which allows prolonged survival under desiccation. Thus, although capa/capaR signaling results in anti-diuresis in *R. prolixus* [42], in flies, the tubule-specific role of capaR in fluid homeostasis modulates desiccation stress responses of the whole organism by limiting fluid loss. Recent work has shown that production of *Drosophila* Short neuropeptide F and tachykinin by specific neurons modulates survival to starvation/desiccation stress [26], so neuropeptides implicated in fluid homeostasis have been identified. We show here that capaR is a canonical GPCR, expressed specifically in the key homeostatic tissue in the fly. This single GPCR directly modulates fluid homeostasis in the whole animal via its ligands, Drm-capa- 1 and -2, and we identify mammalian NMU as a putative functional homolog of these capa peptides.

## Supporting Information

Figure S1 **Validation of capaR RNA interference knockdown.** (**A**) Q-PCR analysis confirmed a 65% decrease in *capaR* mRNA levels in the whole fly compared to control flies. Data are expressed as 10^−5^ ng of *capaR* mRNA ± SEM, *N* = 3.
(TIFF)

Figure S2 **Tubule mRNA expression of** ***GRK-1*** **and** ***GRK-2*** **under capa-1 stimulation.** Wild-type (*Canton-S*) tubules were excised, incubated in Schneider's medium for 3 h as controls, or treated with 10^−7^ M (final concentration) of capa-1 in Schneider's for 3 h. Samples were prepared for Q-PCR to assess *GRK-1* or *GRK-2* expression levels in control and capa-1-treated tubules (shaded bars). Data were normalized against the *rp49* standard, and expressed as ng *GRK-1* or *GRK-2* mRNA ± SEM, *N* = 3.
(TIFF)

Table S1 **Affinities of the** ***Drosophila*** **capa peptides.** (TEV): two-electrode voltage clamp; (CHO): Chinese hamster ovary.
(TIFF)

Video S1 **Translocation of β-arrestin2-eYFP in response to capa-1 in capaR expressing S2 cells.** Video from confocal microscopy series showing the response of S2 cells transiently expressing β-arrestin2-eYFP and capaR constructs to 10^−7^ M capa-1. Within 30 seconds of exposure to capa-1 peptide, the fluorescence translocates to become clearly associated with the membrane.
(AVI)
