# Supplementary figures and images for: Mechanism and Function of Drosophila capa GPCR: A Desiccation Stress-Responsive Receptor with Functional Homology to Human NeuromedinU Receptor
Source: PLoS One. 2012 Jan 11;7(1):e29897. doi: 10.1371/journal.pone.0029897 (PMC3256212; doi:10.1371/journal.pone.0029897)

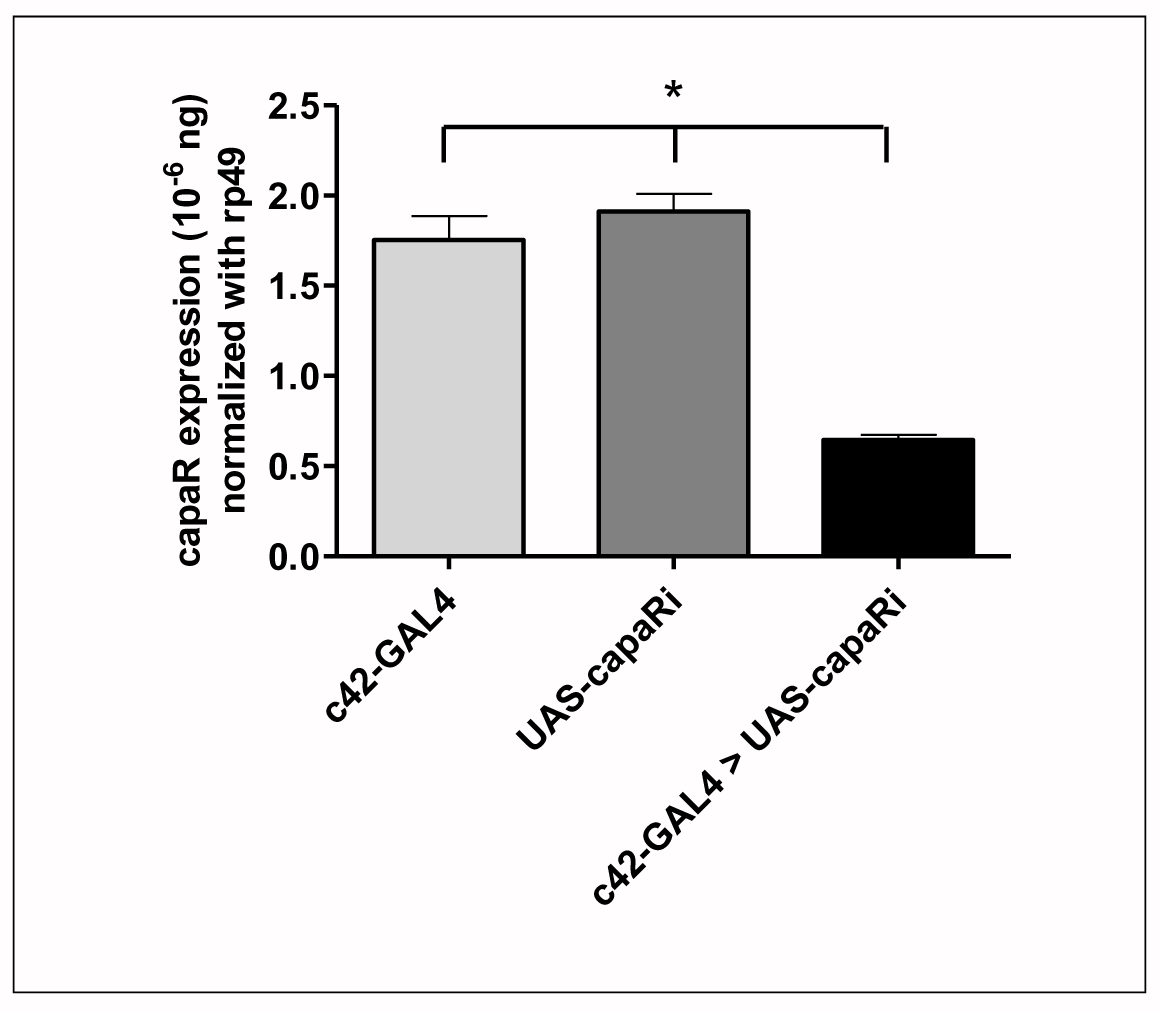

Supplement: Figure S1 — Validation of capaR RNA interference knockdown. (A) Q-PCR analysis confirmed a 65% decrease in capaR mRNA levels in the whole fly compared to control flies. Data are expressed as 10−5 ng of capaR mRNA ± SEM, N = 3. (TIFF) [file pone.0029897.s001.tiff]

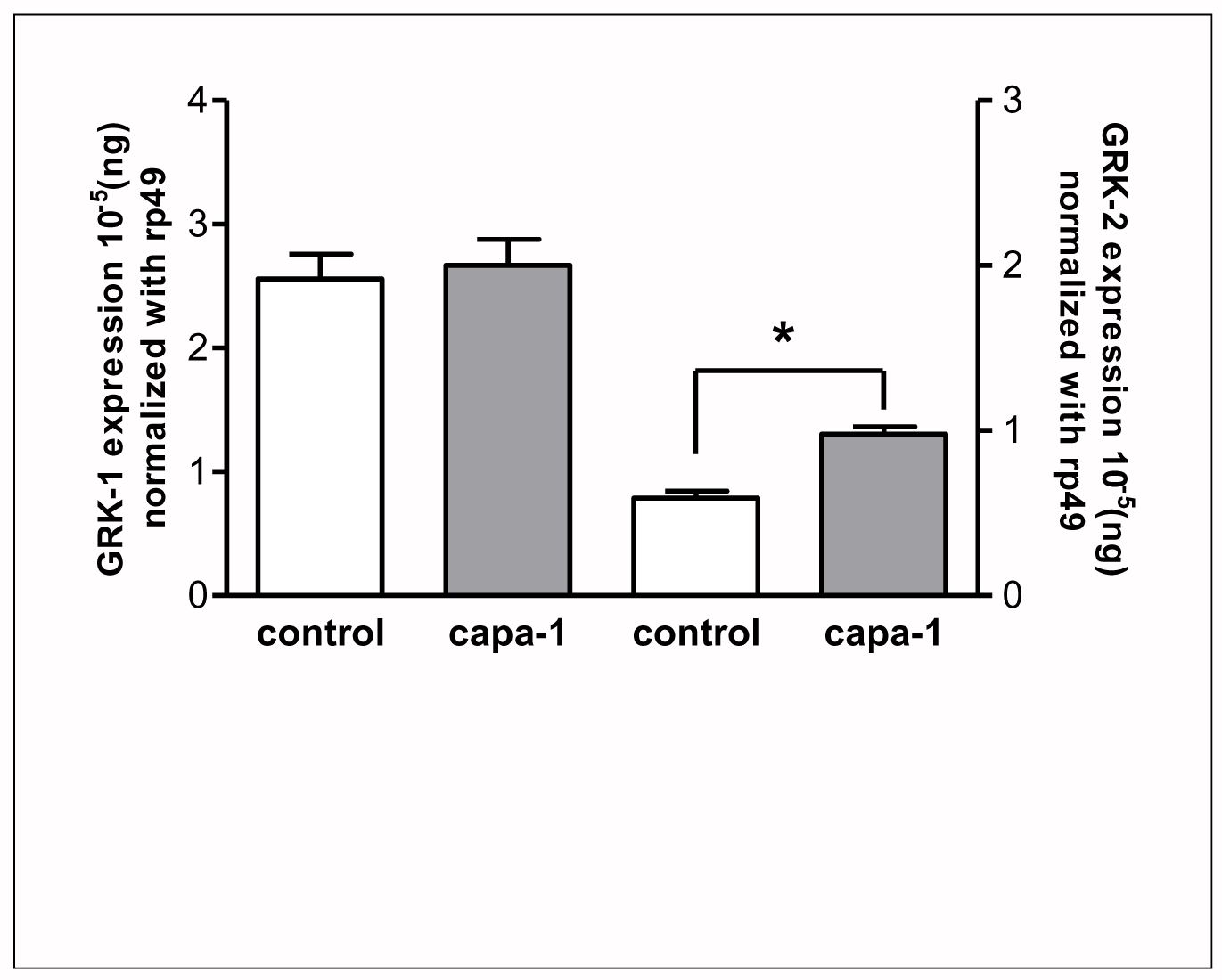

Supplement: Figure S2 — Tubule mRNA expression of GRK-1 and GRK-2 under capa-1 stimulation. Wild-type (Canton-S) tubules were excised, incubated in Schneider's medium for 3 h as controls, or treated with 10−7 M (final concentration) of capa-1 in Schneider's for 3 h. Samples were prepared for Q-PCR to assess GRK-1 or GRK-2 expression levels in control and capa-1-treated tubules (shaded bars). Data were normalized against the rp49 standard, and expressed as ng GRK-1 or GRK-2 mRNA ± SEM, N = 3. (TIFF) [file pone.0029897.s002.tiff]

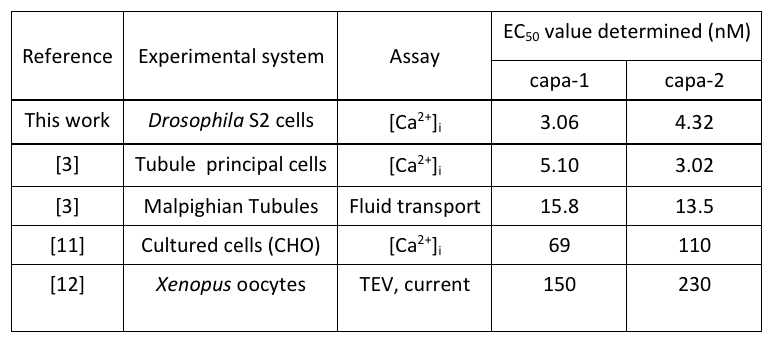

Supplement: Table S1 — Affinities of the Drosophila capa peptides. (TEV): two-electrode voltage clamp; (CHO): Chinese hamster ovary. (TIFF) [file pone.0029897.s003.tiff]
